# Prenatal exposure to ambient air pollutants and early infant growth and adiposity in the Southern California Mother’s Milk Study

**DOI:** 10.1186/s12940-021-00753-8

**Published:** 2021-06-05

**Authors:** William B. Patterson, Jessica Glasson, Noopur Naik, Roshonda B. Jones, Paige K. Berger, Jasmine F. Plows, Hilary A. Minor, Frederick Lurmann, Michael I. Goran, Tanya L. Alderete

**Affiliations:** 1 Department of Integrative Physiology, University of Colorado Boulder, Boulder, CO USA; 2 Department of Pediatrics, The Saban Research Institute, Children’s Hospital of Los Angeles, University of Southern California, Los Angeles, CA USA; 3 Sonoma Technology, Inc, Petaluma, CA USA

**Keywords:** Air pollution, PM_2.5_, Prenatal, Childhood obesity, DOHaD, Infant health

## Abstract

**Background:**

Prior epidemiological and animal work has linked in utero exposure to ambient air pollutants (AAP) with accelerated postnatal weight gain, which is predictive of increased cardiometabolic risk factors in childhood and adolescence. However, few studies have assessed changes in infant body composition or multiple pollutant exposures. Therefore, the objective of this study was to examine relationships between prenatal residential AAP exposure with infant growth and adiposity.

**Methods:**

Residential exposure to AAP (particulate matter < 2.5 and 10 microns in aerodynamic diameter [PM_2.5_, PM_10_]; nitrogen dioxide [NO_2_]; ozone [O_3_]; oxidative capacity [O_x_^wt^: redox-weighted oxidative potential of O_3_ and NO_2_]) was modeled by spatial interpolation of monitoring stations via an inverse distance-squared weighting (IDW2) algorithm for 123 participants from the longitudinal Mother’s Milk Study, an ongoing cohort of Hispanic mother-infant dyads from Southern California. Outcomes included changes in infant growth (weight, length), total subcutaneous fat (TSF; calculated via infant skinfold thickness measures) and fat distribution (umbilical circumference, central to total subcutaneous fat [CTSF]) and were calculated by subtracting 1-month measures from 6-month measures. Multivariable linear regression was performed to examine relationships between prenatal AAP exposure and infant outcomes. Models adjusted for maternal age, pre-pregnancy body mass index, socioeconomic status, infant age, sex, and breastfeeding frequency. Sex interactions were tested, and effects are reported for each standard deviation increase in exposure.

**Results:**

NO_2_ was associated with greater infant weight gain (β = 0.14, *p* = 0.02) and TSF (β = 1.69, *p* = 0.02). PM_10_ and PM_2.5_ were associated with change in umbilical circumference (β = 0.73, *p* = 0.003) and TSF (β = 1.53, *p* = 0.04), respectively. Associations of O_x_^wt^ (p_interactions_ < 0.10) with infant length change, umbilical circumference, and CTSF were modified by infant sex. O_x_^wt^ was associated with attenuated infant length change among males (β = -0.60, *p* = 0.01), but not females (β = 0.16, *p* = 0.49); umbilical circumference among females (β = 0.92, *p* = 0.009), but not males (β = -0.00, *p* = 0.99); and CTSF among males (β = 0.01, *p* = 0.03), but not females (β = 0.00, *p* = 0.51).

**Conclusion:**

Prenatal AAP exposure was associated with increased weight gain and anthropometric measures from 1-to-6 months of life among Hispanic infants. Sex-specific associations suggest differential consequences of in utero oxidative stress. These results indicate that prenatal AAP exposure may alter infant growth, which has potential to increase childhood obesity risk.

**Supplementary Information:**

The online version contains supplementary material available at 10.1186/s12940-021-00753-8.

## Background

In the United States, obesity disproportionately impacts Hispanic youth as nearly 26% of those aged 2–19 have obesity, in contrast to non-Hispanic white (14%) and non-Hispanic Asian (11%) youth [1]. Importantly, obesity in childhood increases the risk for adverse cardiometabolic outcomes in adulthood and premature mortality [2, 3]. Accelerated weight trajectory during the first six months of life has been linked to obesity at three years of age [4] and greater than expected weight gain during any period through four years of age has been found to be associated with increased body mass index (BMI) in adolescence [5]. Recent work supports the hypothesis that developmental programming during the pre-, peri-, and early postnatal periods is sensitive to certain exposures (e.g., maternal nutrition/obesity) that appear to play a critical role in future obesity risk [6]. Exposure to air pollutants during these periods is also suggested as a risk factor for low birth weight, accelerated growth in early life, and/or childhood obesity [7–11]. This may partially explain the aforementioned disparities in childhood obesity, as Hispanics are exposed to higher levels of ambient (AAP) and traffic-related air pollutants [12, 13].

To date, studies examining the link between exposure to air pollutants and weight status have primarily focused on prenatal exposures and birth outcomes (e.g., low/high birth weight, intrauterine growth restriction [IUGR]) [14] or early life/childhood exposures and outcomes in childhood/adolescence [15, 16]. Few studies have examined the influence of prenatal exposure to air pollutants on early infant growth patterns. Greater prenatal exposure to traffic-related air pollution has been shown to result in more rapid infant weight-for-length gain over the first six months of life, particularly among those in the lowest quartile of fetal growth [9] and increased ozone exposure during the 3^rd^ trimester was associated with greater rate of fat mass and adiposity accrual at 5 months old [17]. In other birth cohorts, prenatal exposure to PM_10_ (particulate matter < 10 microns in aerodynamic diameter) was inversely associated with weight-for-age z-score at six months of age [10] while prenatal NO_2_ (nitrogen dioxide) exposure during the 1^st^ trimester was inversely associated both with infant length at 6 months and infant weight at 12 months, which were partially mediated by birth length and birth weight, respectively [8]. These associations between prenatal exposures and postnatal growth patterns may be metabolically programmed via epigenetic changes induced by elevated inflammation and/or oxidative/nitrosative stress [18].

Another important consideration with respect to in utero exposures and both fetal and infant growth patterns is the existence of sex-specific effects [19–21]. For instance, greater prenatal PM_2.5_ (particulate matter < 2.5 microns in aerodynamic diameter) exposure has been linked to lower weight at 2 years of age and beyond in males and an increased weight trajectory in females across the first 6 years of life [11] and greater waist-to-hip ratio among girls and increased fat mass and BMI z-score among boys at 4 years [22]. Two studies examining 1^st^ trimester PM_2.5_ exposure found decreased adiposity among 5-month-old girls [17] and attenuated weight z-scores among pre-school males [23], respectively. Prenatal exposure models in rodents suggest that the potential mechanisms underlying these sexually dimorphic effects may involve differential microglial priming [24], methylation of the promoter region of the leptin gene [25], or altered expression of NPY [26].

As previous studies have been restricted to one pollutant and/or cross-sectional measures of infant growth, there is a need to examine multiple pollutants as well as changes in infant growth parameters over time in order to enable greater insight into the physiological response mechanisms to exposures. In addition, beyond weight and BMI trajectories, there is also a need to examine effects on infant body composition and relative adipose distribution, which are predictive of abdominal adiposity in childhood [27]. Therefore, in the present study, we examined the associations between prenatal residential exposure to ambient air pollutants – PM_2.5_, PM_10_, NO_2_, 24-h ozone (O_3_), and oxidative capacity (O_x_^wt^: a redox-weighted proxy for oxidative potential utilizing NO_2_ and O_3_) – and changes in infant measures of growth and adiposity from 1 to 6 months of life, among Hispanic mother-infant dyads from the Southern California Mother’s Milk Study. We hypothesized that residential exposure to ambient air pollutants during the pregnancy period would be associated with greater change in measures of infant growth and adiposity.

## Methods

### Study population

The longitudinal Mother’s Milk Study is an ongoing cohort of Hispanic mother-infant dyads from Southern California examining growth and development in early life. Recruitment of study participants began in 2016 at Los Angeles County maternity clinics affiliated with the University of Southern California. Maternal inclusion criteria include ≥ 18 years of age at delivery; healthy, term, singleton birth; enrollment by one month postpartum; self-identified Hispanic ethnicity; intention to breastfeed for at least three months postpartum; and ability to read at the 5^th^ grade level in either English or Spanish in order to understand study procedures [28]. Maternal exclusion criteria include medical conditions and/or medications known to potentially affect physical/mental health, nutritional status, or metabolism; tobacco use (defined as > 1 cigarette in the past week); recreational drug use; pre-term/low birth weight; and clinically diagnosed fetal abnormalities. Mothers were recruited to represent an even split of pre-pregnancy BMI class (normal weight [BMI: 18–24.9 kg/m^2^]; overweight [BMI: 25–29.9 kg/m^2^]; obese [BMI: > 30 kg/m^2^]). The Institutional Review Boards of the University of Southern California, Children’s Hospital of Los Angeles, and the University of Colorado Boulder approved study procedures. Written informed consent was obtained from participants prior to enrollment and all analyses in this study.

### Study design

As of July 2020, 212 mother-infant dyads had been enrolled in the study. Maternal medical and family histories were documented at 1 month and infant measures of growth and adiposity were performed at both time points. Information pertinent to relevant covariates were collected, including maternal age, socioeconomic status, infant sex, pre-pregnancy BMI, breastfeeding frequency, and infant age in days. Pre-pregnancy BMI (kg/m^2^) was calculated via weight and height recalled prior to pregnancy. Breastfeeding frequency was ascertained from questionnaires at both time points and was dichotomized to ≥ 7 or < 7 feedings per day at the 6-month timepoint to capture differences over the first 6 months of life since the data was semi-quantitative and this provided a relatively balanced contrast in frequency in this cohort. The Hollingshead Index was utilized to capture socioeconomic status in a standardized fashion [29]. Briefly, this index incorporates four weighted scores consisting of both maternal and paternal educational attainment and occupational prestige, and accounts for marital status and parental living arrangements. Lower scores are indicative of lower SES, while higher scores are indicative of higher SES. Since students, stay-at-home parents, and unemployed persons have no assigned employment categories in the Hollingshead Index, modifications were made to assign these individuals a score of zero (given that these participants were likely to have very little or no income) so as to retain them in the analyses [30]. Alternative adjustment for a poverty index variable (a component of the CalEnviroScreen 3.0 score), representing area-level SES, did not yield appreciable differences in our models (Supplemental Table 3). For the current study, 123 of the 212 mother-infant dyads were included in final our analysis (Supplemental Figure 1). On average, the 123 mothers and infants included did not significantly differ on any important baseline characteristics from those excluded from the current analysis (Supplemental Table 1). Reasons for exclusion consisted of: 1-month clinical visit not completed (n = 3), 6-month clinical visit not yet completed (n = 20), missing air pollution exposure data (n = 24), missing skinfold measures at 6-month visit (n = 1), did not reside at the same address for at least 8 months of the pregnancy period (n = 26), or missing pregnancy address information (n = 4). Additionally, 9 were deemed outliers based on change in infant age (time between study visits) (n = 1), pollutant exposure levels (n = 3), or infant growth change variables (n = 5) as determined by > 3 standard deviations from the mean. Finally, two influential points were removed upon examination of Cook’s distance and residual diagnostics used to meet the assumptions of linear regression analysis.

### Outcome assessment

All outcome measurements were performed by trained clinical research staff. At each visit, infant length was measured in duplicate to the nearest 0.1 centimeter (cm) using an infantometer; infant weight was twice calculated to the nearest 0.1 kilogram (kg) by subtraction of the weight of the mother holding the infant from the weight of the mother alone on a Tanita scale; and infant umbilical circumference was measured to the nearest 0.1 cm in duplicate using a tape measure resistant to stretching. In addition, infant skinfold thickness was measured at both study visits using a commercial caliper at the tricep, subscapular, suprailiac, and midthigh regions. Consistency of the caliper calibration was confirmed with a manufacturer-supplied metal step phantom immediately before its use on each infant. In these measurements, the operator’s thumb and index finger were used to elevate a double fold of skin and subcutaneous tissue in the natural cleavage lines of the skin approximately 1 cm from the site at which the skinfold was measured. For the tricep and midthigh skinfold measures, the corresponding limb was gently extended while the infant was laying down on one side and in the supine position, respectively. The subscapular skinfold was measured with the infant laying on one side at the lower angle of the scapula, in the axis of the skin crease. The suprailiac skinfold was taken immediately above the iliac crest, along the axis of anterior axillary line, with the infant in the supine position. Dynamic skinfold thickness was examined to adjust for tissue waste content by recording each skinfold measurement at exactly 15 s and 60 s with a stopwatch. The sums of the four skinfold measures were calculated to create a proxy for total subcutaneous fat (TSF) at each time point. Further, the sum of the suprailiac and subscapular skinfold thicknesses (representing truncal fat) were calculated and divided by the sum of all four regions to generate a proxy for the ratio of central-to-total subcutaneous fat (CTSF) [27]. For sensitivity analyses, weight-for-age z scores (WAZ) and weight-for-length z scores (WLZ) were also calculated at 1 and 6 months postpartum according to World Health Organization (WHO) standards [31]. Outcome variables representing early infant growth and adiposity were constructed by subtracting 1-month measures from 6-month measures.

### Exposure Assessment

Residential exposure to ambient air pollutants (PM_2.5_, PM_10_, NO_2_, and O_3_) during the pregnancy period was modeled for all 123 mother-infant pairs. PM_2.5_ and PM_10_ were measured in micrograms per cubic meter (μg/m^3^) and NO_2_ and O_3_ were assessed as parts per billion (ppb). O_x_^wt^ was calculated from NO_2_ and O_3_ according to their respective weighted redox potentials [32], as such: [(1.07*NO_2_) + (2.075*O_3_)]/3.145.

Analyses were restricted to participants who had resided at the same address for at least eight months of the pregnancy period to minimize potential for exposure misclassification. Residential address histories were obtained via questionnaire during the baseline study visit and geocoded at the street level utilizing the Texas A&M Geocoder (http://geoservices.tamu.edu/Services/Geocode/). Monthly averages of ambient pollutant exposures were estimated from the U.S. Environmental Protection Agency’s Air Quality System (AQS, http://www.epa.gov/ttn/airs/airsaqs), which records hourly air quality data from ambient monitoring stations. Spatial interpolation of up to four monitoring stations within 50 km of participants’ homes was performed via an inverse distance-squared weighting (IDW2) algorithm. This method has been demonstrated to be robust to leave-one-out validation for the same data source in California [33], with R^2^ values of 0.76, 0.73, 0.53, and 0.46 for O_3_, NO_2_, PM_2.5_, and PM_10_, respectively. Mother-infant pairs were evenly disbursed across Southern California with participants largely clustered in urban centers of Los Angeles, including south-central Los Angeles. Prenatal exposure was modeled based upon the cumulative nine-month average prior to birth. Monthly average exposure estimates for each pollutant were available dating back 12 months from the baseline (1 month postpartum) study visit. Thus, the exposure window spanning from 10 months to 1 month prior to the baseline visit was utilized to capture in utero exposure.

### Statistical analyses

Descriptive statistics are displayed as mean ± standard deviation (SD) for continuous variables and as frequency (%) for categorical variables. Correlations among pollutants were examined using Spearman’s rank tests. Multivariable linear regression analysis was performed to examine the relationship between exposure to residential ambient air pollutants during the pregnancy period and early life changes in infant growth and adiposity. Linear models were adjusted for infant sex, change in infant age (time between visits), pre-pregnancy BMI, breastfeeding frequency at 6 months (dichotomized to ≥ 7 or < 7 times per day), maternal age, and socioeconomic status based on the Hollingshead Index. Two participants were missing information needed for calculating a Hollingshead Index score and were assigned the median value. Levels of ambient air pollutants and infant growth change measures did not differ across tertiles of the Hollingshead Index (Supplemental Table 2). These covariates were chosen based on known associations with infant growth parameters and/or AAP exposure levels [34]. Based upon previous literature [11], infant sex was also examined as a potential effect modifier of the relationship between ambient air pollution and early infant growth. Sensitivity analyses were also performed, which additionally adjusted for birth weight and the baseline variable of each change outcome. However, these results should be interpreted with caution as birth weight may be on the causal pathway [8, 35] and inclusion of baseline values may be an over-adjustment [36]. Also, we did not have detailed information on gestational age (i.e., last menstrual period or physician’s assessment), which adds important context to birth weight [37]. Effect estimates in our single-pollutant models were scaled to one SD of exposure (PM_2.5_ [SD = 1.16 μg/m^3^], PM_10_ [SD = 3.58 μg/m^3^], NO_2_ [SD = 2.39 ppb], O_3_ [SD = 2.48 ppb], O_x_^wt^ [SD = 1.08]). Due to the high correlation among pollutants (Table 2), multipollutant models were not examined. Generalized additive models (GAM) with penalized spline smooth terms were fitted and we visually assessed plotted splines to determine linearity of exposure-outcome associations. Overall, we found some evidence for non-linear associations between prenatal PM_10_ exposure and change in umbilical circumference (pGAM = 0.02) as well as PM_2.5_ and change in total subcutaneous fat (pGAM = 0.04). For these models, we fitted cubic splines to investigate the non-linear associations and subsequently created tertiles to examine the non-linear dose-response relationships. All analyses were conducted with RStudio (Version 1.2.1335; RStudio, Inc.) and figures were made in Prism (Version 8.41; GraphPad Software, Inc.). Statistical significance was defined as a two-sided *p*-value less than 0.05. For interaction terms, statistical significance was defined as a two-sided *p*-value less than 0.1 by virtue of the quantified gain in power given our sample size and variables of interest [38]. Effect estimates are reported with 95% confidence intervals (CIs).

## Results

Maternal and infant characteristics at 1 and 6 months are shown in Table 1. On average, mothers were 29.2 years old at baseline, and prior to pregnancy, 31 (25.2%) mothers were classified as normal weight, 48 (39.0%) as overweight, and 44 (35.8%) as obese. The proportion of women breastfeeding decreased from 99.2% at baseline to 76.4% at six months (*p* < 0.001). Approximately 42.3% of infants were male and the average birth weight was 3.4 kg (range: 2.3 to 4.5 kg). As expected, all infant growth parameters increased from 1 to 6 months of age (p_all_ < 0.01). During the pregnancy period, mean residential exposure to PM_2.5_ and PM_10_ ranged between 10.6 and 14.6 μg/m^3^ and 25.3 and 38.3 μg/m^3^, respectively. For NO_2_, O_3_, and O_x_^wt^, exposure levels ranged between 12.6 and 22.9 ppb, 22.1 and 31.2 ppb, and 21.3 and 25.8, respectively. Further, exposures to ambient air pollutants were significantly correlated during the pregnancy period (Table 2).

**Table 1:** Characteristics of Mother-Infant Dyads from the Mother’s Milk Study at 1 and 6 Months Postpartum

	**1 Month** **Mean ± SD**	**6 Months** **Mean ± SD**
**Maternal Characteristics**		
Maternal Age (years)	29.23 ± 6.00	29.64 ± 6.11
Maternal BMI (kg/m^2^)	30.27 ± 4.86	30.88 ± 5.52
Breastfeeding (% yes)	99.2%	78.9%
Breastf Freq (% ≥ 7)	70.7%	27.6%
**Infant Characteristics**		
Age (days)	32.35 ± 3.11	185.11 ± 8.10
Infant Sex (% male)	42.3%	
Weight (kg)	4.63 ± 0.45	8.07 ± 0.77
Length (cm)	54.36 ± 1.85	67.14 ± 2.20
**Infant Anthropometrics**		
Umbilical Circumference (cm)	36.05 ± 2.12	41.70 ± 2.69
Total Subcutaneous Fat (mm)	29.75 ± 5.03	42.86 ± 7.81
Central:Total Subc Fat	0.39 ± 0.04	0.32 ± 0.04
Midthigh Skinfold (mm)	11.57 ± 2.27	20.49 ± 4.13
Tricep Skinfold (mm)	6.50 ± 1.50	8.68 ± 2.35
Suprailiac Skinfold (mm)	4.37 ± 1.23	5.87 ± 1.65
Subscapular Skinfold (mm)	7.31 ± 1.68	7.81 ± 1.96

1- and 6-month characteristics of 123 Hispanic mother-infant dyads from the Southern California Mother’s Milk Study. Data are reported mean and standard deviation (SD) unless otherwise noted. Breastfeeding frequency at 6 months of age is shown dichotomized as ≥ 7 or < 7 times per day. Total subcutaneous fat is the sum of the four infant skinfold thickness measures in millimeters. The Central:Total subcutaneous fat variable is the sum of the suprailiac and subscapular skinfold thicknesses divided by all four skinfold measures

**Table 2:** Residential Prenatal Exposures to Ambient Air Pollutants

Pollutant	Mean ± SD	PM_2.5_ (μg/m^3^)	PM_10_ (μg/m^3^)	NO_2_ (ppb)	O_3_ (ppb)	O_x_^wt^
PM_2.5_ (μg/m^3^)	11.95 ± 1.15	1				
PM_10_ (μg/m^3^)	31.03 ± 3.54	0.75***	1			
NO_2_ (ppb)	18.05 ± 2.34	0.47***	0.27**	1		
O_3_ (ppb)	26.02 ± 2.38	-0.17	0.18*	-0.80***	1	
O_x_^wt^	23.30 ± 1.04	0.12	0.51***	-0.45***	0.88***	1

Mean and standard deviations of exposure levels and Spearman’s rank correlation between residential ambient air pollutants during the prenatal period. Correlation coefficients (ρ) and *p*-values are shown for each pair of exposures. ***p < 0.001, **p < 0.01, *p < 0.05. PM_2.5_ and PM_10_ were measured in micrograms per cubic meter and NO_2_ and O_3_ were measured in parts per billion. O_x_^wt^ was calculated by the weighted redox potential of NO_2_ and O_3_, as follows: ([1.07*NO_2_ + 2.075*O_3_]/3.145)

PM_2.5_ and PM_10_ were statistically significantly associated with increased measures of infant body composition (Table 3). For example, PM_2.5_ was associated with a larger increase in TSF (β = 1.53; CI: 0.11, 2.94; *p* = 0.04); however, there was some evidence for non-linearity (pGAM = 0.04). Therefore, we examined this association across tertiles of exposure and observed a positive association in the lowest tertile of exposure (β = 15.2; *p* = 0.03) and smaller inverse associations in the second (β = -2.2; *p* = 0.84) and third tertiles (β = -1.6; *p* = 0.43), which did not reach statistical significance. PM_10_ exposure was positively associated with change in TSF (β = 1.40; CI: -0.03, 2.82; *p* = 0.05). Additionally, PM_10_ (β = 0.73; CI: 0.25, 1.21; *p* = 0.003) was positively associated with greater changes in umbilical circumference from 1 to 6 months of age, which also showed evidence for non-linearity (pGAM = 0.02). Across tertiles of PM_10_ exposure, this association was positive in the lowest tertile of exposure (β = 2.37; *p* = 0.004) and modestly inversely associated in the second (B = -0.26; *p* = 0.37) and third tertiles (β = -0.10; *p* = 0.73) but these associations did not reach statistical significance. Overall, results were largely consistent with additional adjustment for infant birth weight or baseline measures of each outcome (Supplemental Figure 2). Lastly, the associations between PM exposure and measures of infant growth and body composition were not modified by infant sex (P_interactions_ > 0.10) (Table 4).

**Table 3:** Prenatal Exposures and Infant Changes from 1- to 6-Months Postpartum

Exposure	Δ Outcome	β	95% CIs	*P*-value
PM_2.5_	Weight	0.06	-0.06, 0.17	0.34
	Length	-0.04	-0.36, 0.28	0.80
	Umbilical Circ	0.47	-0.02, 0.96	0.06
	TSF	1.53	0.11, 2.94	0.04
	CTSF	0.00	-0.01, 0.01	0.82
PM_10_	Weight	0.07	-0.05, 0.18	0.27
	Length	0.07	-0.26, 0.39	0.68
	Umbilical Circ	0.73	0.25, 1.21	0.003
	TSF	1.40	-0.03, 2.82	0.05
	CTSF	0.01	-0.00, 0.01	0.22
NO_2_	Weight	0.14	0.02, 0.25	0.02
	Length	0.23	-0.09, 0.56	0.16
	Umbilical Circ	0.25	-0.25, 0.76	0.32
	TSF	1.69	0.25, 3.12	0.02
	CTSF	-0.00	-0.01, 0.00	0.37
O_3_	Weight	-0.10	-0.22, 0.02	0.09
	Length	-0.20	-0.53, 0.13	0.23
	Umbilical Circ	0.26	-0.25, 0.77	0.31
	TSF	-1.11	-2.57, 0.35	0.14
	CTSF	0.01	0.00, 0.02	0.07
O_x_^wt^	Weight	-0.05	-0.17, 0.07	0.41
	Length	-0.13	-0.46, 0.20	0.45
	Umbilical Circ	0.59	0.09, 1.09	0.02
	TSF	-0.37	-1.85, 1.11	0.62
	CTSF	0.01	0.00, 0.02	0.04

Multivariable linear regression was performed to examine the relationships between prenatal exposure to ambient air pollutants and changes in infant growth from 1 to 6 months of age. Beta coefficients and 95% confidence intervals are shown for a one standard deviation increase in exposure (PM_2.5_ [SD = 1.15 μg/m^3^], PM_10_ [SD = 3.54 μg/m^3^], NO_2_ [SD = 2.34 ppb], O_3_ [SD = 2.38 ppb], O_x_^wt^ [SD = 1.04]). Multivariable linear models adjust for infant sex, infant age, pre-pregnancy BMI, breastfeeding frequency, maternal age, and socioeconomic status. TSF and CTSF represent changes in total subcutaneous fat and the ratio of central to total subcutaneous fat, respectively

**Table 4:** Changes in Infant Growth Measures from 1 to 6 Months Postpartum by Infant Sex

	**Females**			**Males**			
**PM**_**2.5**_** and Δ Outcome**	**β**	**95% CIs**	***P*-value**	**β**	**95% CIs**	***P*-value**	**P interaction**
Weight	0.01	-0.15, 0.17	0.92	0.15	-0.03, 0.33	0.09	0.25
Length	-0.15	-0.61, -0.27	0.45	0.16	-0.32, 0.65	0.50	0.34
Umbilical Circumference	0.35	-0.32, 1.03	0.30	-0.74	-0.03, 1.50	0.06	0.43
TSF	1.52	-0.35, 3.38	0.11	1.45	-0.97, 3.87	0.23	0.96
CTSF	0.00	-0.01, 0.01	0.60	0.00	-0.02, 0.01	0.79	0.70
**PM**_**10**_** and Δ Outcome**	**β**	**95% CIs**	***P*-value**	**β**	**95% CIs**	***P*-value**	**P interaction**
Weight	0.02	-0.14, 0.18	0.81	0.14	-0.03, 0.32	0.11	0.35
Length	0.09	-0.36, 0.54	0.69	0.05	-0.44, 0.54	0.84	0.77
Umbilical Circumference	0.72	0.04, 1.39	0.04	0.80	0.04, 1.56	0.04	0.99
TSF	1.40	-0.51, 3.31	0.15	1.29	-1.14, 3.72	0.29	0.98
CTSF	0.00	-0.01, 0.01	0.64	0.01	-0.01, 0.02	0.24	0.36
**NO**_**2**_** and Δ Outcome**	**β**	**95% CIs**	***P*-value**	**β**	**95% CIs**	***P*-value**	**P interaction**
Weight	0.13	-0.04, 0.29	0.13	0.18	0.01, 0.36	0.04	0.53
Length	0.14	-0.33, 0.61	0.56	0.37	-0.10, 0.84	0.12	0.43
Umbilical Circumference	0.07	-0.66, 0.80	0.84	0.71	-0.05, 1.46	0.07	0.16
TSF	1.38	-0.63, 3.39	0.17	2.42	0.10, 4.73	0.04	0.43
CTSF	-0.00	-0.01, 0.01	0.75	-0.00	-0.02, 0.01	0.63	0.80
**O**_**3**_** and Δ Outcome**	**β**	**95% CIs**	***P*-value**	**β**	**95% CIs**	***P*-value**	**P interaction**
Weight	-0.11	-0.27, 0.06	0.20	-0.15	-0.34, 0.03	0.10	0.76
Length	0.04	-0.43, 0.51	0.87	-0.60	-1.07, -0.13	0.01	0.05
Umbilical Circumference	0.57	-0.13, 1.27	0.11	-0.39	-1.21, 0.43	0.34	0.05
TSF	-1.02	-3.01, 0.96	0.31	-1.64	-4.13, 0.85	0.19	0.72
CTSF	0.00	-0.01, 0.01	0.56	0.01	-0.00, 0.02	0.10	0.18
**O**_**x**_^**wt**^** and Δ Outcome**	**β**	**95% CIs**	***P*-value**	**β**	**95% CIs**	***P*-value**	**P interaction**
Weight	-0.07	-0.23, 0.10	0.43	-0.08	-0.27, 0.11	0.41	0.91
Length	0.16	-0.31, 0.63	0.49	-0.60	-1.07, -0.12	0.01	0.02
Umbilical Circumference	0.92	0.24, 1.61	0.01	-0.00	-0.83, 0.82	0.99	0.06
TSF	-0.54	-2.55, 1.48	0.60	-0.47	-3.02, 2.08	0.71	0.88
CTSF	0.00	-0.01, 0.01	0.51	0.01	0.00, 0.03	0.03	0.09

Beta coefficients and 95% confidence intervals are shown for a one standard deviation increase in exposure (PM_2.5_ [SD = 1.15 μg/m^3^], PM_10_ [SD = 3.54 μg/m^3^], NO_2_ [SD = 2.34 ppb], O_3_ [SD = 2.38 ppb], O_x_^wt^ [SD = 1.04]) via multivariable linear regression. Results are reported for females and males with the corresponding *p*-value for the interaction between O_x_^wt^ and infant sex. All stratified models adjusted for infant age, pre-pregnancy BMI, breastfeeding frequency, maternal age, and socioeconomic status

Higher exposure to NO_2_ during the pregnancy period was associated with a greater increase in infant weight (β = 0.14; CI: 0.02, 0.25; *p* = 0.02) and TSF (β = 1.69; CI: 0.25, 3.12; *p* = 0.02) from 1 to 6 months of life. O_x_^wt^ was associated with change in umbilical circumference (β = 0.59; CI: 0.09, 1.09; *p* = 0.02) and an increased CTSF (β = 0.01; CI: 0.00, 0.02; *p* = 0.04) from 1 to 6 months of age, and infant sex was found to modify the effect of these relationships (p_interactions_ < 0.1) (Table 4). Specifically, O_x_^wt^ was associated with change in umbilical circumference among females (β = 0.92; CI: 0.24, 1.61; *p* = 0.009), but not males (β = -0.00; CI: -0.83, 0.82; *p* = 0.99), and change in CTSF among males (β = 0.01; CI: 0.00, 0.03; *p* = 0.03), but not females (β = 0.00; CI: -0.01, 0.01; *p* = 0.51). O_x_^wt^ and O_3_ were not associated with change in infant length (β = -0.13; CI: -0.46, 0.20; *p* = 0.45 and β = -0.20; CI: -0.53, 0.13; *p* = 0.23, respectively), but infant sex was found to modify the effect of these relationships (P_interactions_ = 0.06). Specifically, O_x_^wt^ and O_3_ were each inversely associated with infant length change (β = -0.60; CI: -1.07, -0.12; *p* = 0.01 and β = -0.60; CI: -1.07, -0.13; *p* = 0.01, respectively) among males, but not females (β = 0.16; CI: -0.31, 0.63; *p* = 0.49 and β = 0.04; CI: -0.43, 0.51; *p* = 0.87, respectively). These associations were largely robust to further adjustments for birth weight and baseline values, respectively (Supplemental Figure 2). Lastly, we conducted a sensitivity analysis by examining changes in infant z-scores from 1 to 6 months and similarly found that prenatal AAP was associated with greater increases in some standardized measures of infant body composition (Supplemental Table 4).

## Discussion

In this study of Hispanic mother-infant dyads from the Mother’s Milk Study cohort, prenatal ambient air pollution exposure was associated with increased postnatal growth and adiposity among infants from 1 to 6 months of life. Higher in utero exposure to NO_2_ was associated with greater increases in infant weight and total subcutaneous fat, while PM_10_ and PM_2.5_ were associated with larger increases in umbilical circumference and total subcutaneous fat, respectively. Upon examination of non-linearity, positive associations of particulate matter with infant body composition appeared to plateau beyond the first tertile of exposure. Consistent with previous studies [11, 22], we observed sex-specific findings. O_x_^wt^ was associated with change in umbilical circumference and a modestly larger increase in CTSF, with these associations observed only among females and males, respectively, following stratification by sex. Likewise, O_x_^wt^ and O_3_ exposure were inversely associated with change in infant length only among males. Overall, greater AAP exposure was associated with increased adiposity (i.e., TSF, umbilical circumference), but there was only one modest main effect for increased CTSF, which suggests that AAP may influence total adiposity while not favoring central adiposity to other fat depots at this life stage.

The potential molecular mechanisms underlying the associations between prenatal exposure to air pollutants [39] and altered postnatal phenotypes may involve formation of DNA adducts [40], mitochondrial oxidative stress [41, 42], telomere shortening [43, 44], polymorphisms of detoxification enzymes [45, 46], global hypomethylation [47], placental microRNA expression [48], and/or aryl hydrocarbon hydroxylase activity [49]. Upon exposure to particulate matter, several of these mechanisms may converge, directly and indirectly, to induce oxidative/nitrosative stress [50, 51] and affect growth patterns through epigenetic changes [18] and increased inflammation [52]. For example, greater exposure to PM_2.5_ during the second trimester of pregnancy has been shown to be associated with reduced methylation of the placental leptin promoter [53] and PM_10_ exposure during the first and second trimesters has been associated with methylation of genes regulating fetal growth and glucocorticoid metabolism [54]. Furthermore, exposure to air pollutants could influence early infant growth via alterations to the developing gut microbiome [55, 56]. It remains to be determined if air pollutants can physically traverse the placenta in humans, but recent work has identified black carbon particles at the fetal side of the human placenta [57] and a rabbit model found aggregates of nanoparticles in trophoblastic cells and fetal blood upon maternal exposure to diesel exhaust [58].

Findings from the INMA (Infancia y Medio Ambiente [Environment and Childhood]) cohort included an inverse association (trend) between prenatal NO_2_ exposure and length z-score at 6 months of age, particularly during the first trimester [8]. Although we did not find an association with NO_2_ and change in length, we observed attenuated growth in length from 1 to 6 months of age among males exposed to greater prenatal O_x_^wt^, which incorporates both NO_2_ and O_3_. O_x_^wt^ acts as a proxy for the potential to induce oxidative stress. Indeed, previous work in newborn twins has shown that males have higher levels of 15-F_2t_-isoprostane, a marker for oxidative stress, in both like-sex and unlike sex pairs [59], indicating males may be particularly susceptible to in utero oxidative stress. This finding also coheres with the INMA study which found that placental mitochondrial DNA content, which is notably susceptible to oxidative stress, partially mediated the inverse relationship between prenatal NO_2_ exposure and length at 6 months [8]. Of relevance, restricted height attainment appears to account for the association of greater childhood BMI in those exposed in utero to maternal smoking [60].

Many, but not all [14], prenatal exposure studies have observed intrauterine growth restriction or low birth weight in response to higher exposure to air pollutants, and this effect has been hypothesized to be due to compromised transfer of nutrients from the mother to the developing fetus [61]. This is in line with the thrifty phenotype hypothesis, which postulates that cues from the environment during development can potentiate fetal reprogramming and alter growth trajectories, including “catch-up/rapid growth”, and increase future risk for metabolic diseases [62–64]. Findings from the Project Viva cohort supported the catch-up growth hypothesis, wherein those with the highest exposure to traffic density and black carbon had higher odds for the phenotype of lower fetal growth (birth weight) and more rapid 0 to 6 month weight-for-length gain [9]. In the current study, we did not observe an association between prenatal exposure and birth weight; however, we are limited to drawing any firm conclusions due to incomplete information on gestational age (i.e., we did not have date of last menstrual period or physician’s assessment of gestational age), which adds important context to birth weight and risk for future disease [37]. We did, however, find a similar pattern of increased rate of growth and adiposity measures from 1 to 6 months of life among those exposed to higher levels of NO_2_ and PM. Longitudinal examination of weight trajectory in response to prenatal PM_2.5_ in the Children’s HealthWatch cohort found consistently higher weight across the first 6 years of life among females with above median exposure [11]. This is in line with our finding of prenatal particulate matter exposure and increased rate of change in umbilical circumference and total subcutaneous fat, both measures of adiposity, and similarly, greater change in umbilical circumference among females in response to O_x_^wt^ exposure. Our findings specific to prenatal PM_10_ exposure were similar to those with PM_2.5_ and each showed evidence of non-linear dose–response effects, which could be attributable to a saturation point [65, 66] beyond the first tertile of PM exposure for these specific relationships. This could be due to a confluence of molecular mechanisms that impart epigenetic programming with an upper threshold of exposure [18]. In contrast, the Mothers and Children’s Environmental Health cohort found an inverse association with prenatal PM_10_ and weight-for-age z score at 6 months of age [10]. However, this effect did not persist at any other intervals up to age 5 years [10].

Whether early growth patterns linked to prenatal air pollutant exposure persist into adolescence and young adulthood remains to be determined. While prenatal exposure has been linked to slower fetal growth and lower birth weight, both low and high birth weight have been observed to predict a ‘stable obesity’ growth trajectory through childhood [6]. In addition, a similarly sized ‘later-onset obesity’ group has been identified that showed a high and earlier likelihood of obesity by 2 years of age and exhibited similar developmental programming risk factors [6], which may be reflective of subtle epigenetic changes that occur during the gestational period that increase susceptibility to exposures later in life [67]. The ACCESS (Asthma Coalition on Community, Environment and Social Stress) cohort found early and mid-pregnancy exposure to PM_2.5_ to be associated with fat mass and BMI z-score among boys and mid-pregnancy exposure with increased waist-to-hip ratio among girls, respectively, at 4 years old [22]. Additionally, the Mothers and Children Study found greater prenatal exposure to polycyclic aromatic hydrocarbons to be associated with higher BMI z-scores at ages 5 and 7 years [68]. Another cohort from Wuhan, China found that prenatal NO_2_ exposure, particularly in the 3^rd^ trimester, was associated with childhood obesity at 2 years old and a faster growth in childhood BMI-for-age z-score [7]. In contrast, Project Viva found no association between prenatal exposure to traffic-related air pollution and BMI trajectory, including the timing and magnitude of BMI peak in infancy and adiposity rebound [69], both important predictors of obesity later in life [70]. This discrepancy may have been attributable to differences in race/ethnicity, maternal obesity, and breastfeeding practices from other cohorts [69].

This study should be interpreted with the context of several strengths and limitations. In contrast to previous relevant work, we examined the effects of multiple pollutant exposures (PM_2.5_, PM_10_, NO_2_, O_3_, O_x_^wt^). This aids both in identifying the unique effects of different pollutants and distinguishing the exposures of greatest concern for a given outcome, which can help direct effective policy interventions. However, the high degree of correlation between pollutants, particularly NO_2_ and O_3_, which are known to be intimately related via their atmospheric photochemistry [32], may limit interpretability of their individual effects in this study. Also, despite being strongly inversely correlated with one another, O_3_ and NO_2_ were both positively associated with umbilical circumference (although neither model was statistically significant and both confidence intervals also included inverse associations). This may have been due to our relatively small exposure contrasts for these pollutants. Despite this, our additional use of O_x_^wt^, the redox-weighted oxidative variable utilizing NO_2_ and O_3_, alternatively enabled investigation into their joint effects. The longitudinal study design allowed for examination of temporal exposure-outcome relationships and persistence of effects over time that cannot be captured cross-sectionally. All participants were of Hispanic ethnicity; while this may limit generalizability of study findings, this group is often exposed to higher levels of air pollutants and at greater risk for childhood obesity. Misclassification of exposure could have occurred due to either time spent away from residences or discordant indoor exposures; however, any misclassification likely would have biased effects towards the null [71]. The IDW2 interpolation exposure assessment method in this analysis is limited by spatial resolution relative to other exposure assessment methods like land-use-regression or hybrid models but is justified by the focus on associations with regional air pollutant levels in the present analysis. Furthermore, prior work by our group has shown that the IDW2 method in California has little bias on average (0.1 ppb, 0.7 ppb, 0.4 µg/m^3^, and 0.5 µg/m^3^ for O_3_, NO_2_, PM_2.5_, and PM_10_), and acceptable error (± 15%, 34%, 31%, and 35% for O_3_, NO_2_, PM_2.5_, and PM_10_) [33]. While our study had a relatively small sample size, the availability of information regarding important covariates and detailed phenotyping of mothers and infants allowed for adjustment of known confounders in several model iterations. In addition to traditional measures of growth, we included several measures of infant body composition enabling greater insight into infant growth. The use of a skinfold caliper to measure body composition (in place of DEXA) could be considered a limitation, but skinfold thickness may be a reasonable proxy for DEXA in this age group [72] and is positively associated with total and abdominal fat at six years of age [27]. Incomplete information with respect to date of conception and gestational age prevent us from adjusting for this in our statistical models, yet models with and without birth weight did not appear to appreciably influence the results. Future investigations in the Mother’s Milk Study will include both pregnancy and early life exposures to air pollutants through 36 months of age, which will be used to further investigate the effects of exposure to air pollutants on rapid infant growth and risk for childhood obesity.

## Conclusion

Prenatal ambient air pollution exposure was associated with increased weight gain and anthropometric measures from 1 to 6 months of life in 123 Hispanic infants from Southern California. Sex-specific associations suggest differential consequences of in utero oxidative stress. These results indicate that prenatal ambient air pollution exposure may alter infant growth, which has potential to increase childhood obesity risk. Interventions to address the obesity and non-communicable disease epidemics should consider personal and policy-based strategies for reducing exposure during critical periods in early life.

## Supplementary Information

**Additional file 1: Supplemental Table 1.** Mother-Infant Dyads were Similar to those Excluded from the Current Analysis. 1-month characteristics of Hispanic mother-infant dyads from the Southern California Mother’s Milk Study are shown for both those included and those excluded from the current study. Data are reported mean and standard deviation (SD) unless otherwise noted. Group means of continuous variables were compared via two-sample *t*-tests and binary variables were compared via Chi-square test. Breastfeeding frequency at 6 months of age is shown dichotomized as ≥ 7 or < 7 times per day. Total subcutaneous fat is the sum of the four infant skinfold thickness measures in millimeters. The Central:Total subcutaneous fat variable is the sum of the suprailiac and subscapular skinfold thicknesses divided by all four skinfold measures.

**Additional file 2: Supplemental Table 2.** Levels of Ambient Air Pollutant Exposures and Infant Growth Outcomes Across Tertiles of Socioeconomic Status. Means and standard deviations of ambient air pollutant exposure levels and infant growth change variables are presented across tertiles of socioeconomic status (Hollingshead Index). Tertile means of continuous variables were compared via one-way ANOVA and *p*-values are shown. TSF represents the change in infant total subcutaneous fat, which is the sum of the four infant skinfold thickness measures in millimeters. CTSF represents the change in infant central:total subcutaneous, which is the sum of the suprailiac and subscapular skinfold thicknesses divided by all four skinfold measures. O_x_^wt^ is the redox-weight oxidative potential of NO_2_ and O_3_.

**Additional file 3: Supplemental Table 3.** Sensitivity Analysis: Prenatal Exposures and Infant Changes from 1- to 6-Months Postpartum with Adjustment for Area-Level Socioeconomic Status. Multivariable linear regression was performed to examine the relationships between prenatal exposure to ambient air pollutants and changes in infant growth from 1 to 6 months of age with adjustment for a poverty index variable in place of the individual-level socioeconomic status variable in the main model. Beta coefficients and 95% confidence intervals are shown for a one standard deviation increase in exposure (PM_2.5_ [SD = 1.15 μg/m^3^], PM_10_ [SD = 3.54 μg/m^3^], NO_2_ [SD = 2.34 ppb], O_3_ [SD = 2.38 ppb], O_x_^wt^ [SD = 1.04 ppb]). Multivariable linear models adjust for infant sex, infant age, pre-pregnancy BMI, breastfeeding frequency, maternal age, and poverty index (a component of the CalEnviroScreen 3.0 score). TSF and CTSF represent changes in total subcutaneous fat and the ratio of central to total subcutaneous fat, respectively.

**Additional file 4: Supplemental Table 4.** Sensitivity Analysis: Prenatal Exposures and Infant Z-Score Changes from 1- to 6-Months Postpartum. Table displays the results for multivariable linear regression models examining prenatal ambient air pollutant exposure and changes in infant z-score measures (weight-for-age and weight-for-length). Beta coefficients are shown for a one standard deviation increase in exposure (PM_2.5_ [SD = 1.15 μg/m^3^], PM_10_ [SD = 3.54 μg/m^3^], NO_2_ [SD = 2.34 ppb], O_3_ [SD = 2.38 ppb], O_x_^wt^ [SD = 1.04]). Models adjusted for pre-pregnancy BMI, breastfeeding frequency, maternal age, and socioeconomic status.

**Additional file 5: Supplemental Figure 1.** Flowchart Detailing Participants Retained for the Present Analysis. At the time of our analyses, 212 participants had been enrolled in the Mother’s Milk Study. There were 24 participants who had completed both clinical study visits but did not yet have ambient pollutant exposure estimates available. Among the 5 change in infant growth variable outliers, 2 were outliers from infant length change, 1 was an outlier from infant weight change, 1 was an outlier from umbilical circumference change, and 1 was from change in CTSF.

**Additional file 6: Supplemental Figure 2.** Sensitivity Analysis: Multivariable Linear Models Additionally Adjusted for Birth Weight and Baseline of Change Variables. Fully adjusted models controlled for infant sex, infant age, pre-pregnancy BMI, breastfeeding frequency, maternal age, and socioeconomic status (effect estimates denoted by circles). Sensitivity analyses examining multivariable linear regression models additionally adjusting for birth weight (effect estimates denoted by squares) or the baseline value of the change variable (effect estimates denoted by black triangles), respectively, were performed to further evaluate the relationships between prenatal exposure to ambient air pollutants and changes in infant growth from 1 to 6 months of age. Beta coefficients and 95% confidence intervals are shown for a one standard deviation increase in exposure (PM_2.5_ [SD = 1.15 μg/m^3^], PM_10_ [SD = 3.54 μg/m^3^], NO_2_ [SD = 2.34 ppb], O_3_ [SD = 2.38 ppb], O_x_^wt^ [SD = 1.04]).

**Additional file 7: Supplemental Figure 3.** Prenatal NO_2_ and O_3_ were Associated with a Greater Change in Umbilical Circumference. Scatterplots display infant change in umbilical circumference in relation to prenatal NO_2_ and O_3_, respectively. Plots were made to visualize the distribution of these data due to a strong inverse correlation between NO_2_ and O_3_, yet similar direction of effect.

## Data Availability

The datasets used and/or analyzed during the current study are available from the corresponding author on reasonable request.

## Abbreviations

PM_2.5_: Fine particulate matter
PM_10_: Coarse particulate matter
NO_2_: Nitrogen dioxide
O_3_: Ozone
O_x_^wt^: Redox-weighted oxidative capacity
TSF: Total subcutaneous fat
CTSF: Central:total subcutaneous fat
AAP: Ambient air pollutants
BMI: Body mass index
