# Supplementary material for: Prenatal exposure to ambient air pollutants and early infant growth and adiposity in the Southern California Mother’s Milk Study
Source: Environ Health. 2021 Jun 5;20:67. doi: 10.1186/s12940-021-00753-8 (PMC8180163; doi:10.1186/s12940-021-00753-8)
Supplement: Supplementary file 1 — Additional file 1: Supplemental Table 1. Mother-Infant Dyads were Similar to those Excluded from the Current Analysis. 1-month characteristics of Hispanic mother-infant dyads from the Southern California Mother’s Milk Study are shown for both those included and those excluded from the current study. Data are reported mean and standard deviation (SD) unless otherwise noted. Group means of continuous variables were compared via two-sample t-tests and binary variables were compared via Chi-square test. Breastfeeding frequency at 6 months of age is shown dichotomized as ≥ 7 or < 7 times per day. Total subcutaneous fat is the sum of the four infant skinfold thickness measures in millimeters. The Central:Total subcutaneous fat variable is the sum of the suprailiac and subscapular skinfold thicknesses divided by all four skinfold measures. [file 12940_2021_753_MOESM1_ESM.docx]

**Supplemental Table 1. Mother-Infant Dyads were Similar to those Excluded from the Current Analysis**

|  | **1 Month**  ***Mean ± SD*** | **1 Month**  ***Mean ± SD*** | **P-value** |
| --- | --- | --- | --- |
|  | **Included (n=123)** | **Excluded (n=73)** |  |
| Maternal Characteristics |  |  |  |
| Maternal Age (years) | 29.23 ± 6.00 | 28.23 ± 6.35 | 0.27 |
| Maternal BMI (kg/m^2^) | 30.27 ± 4.86 | 30.46 ± 5.79 | 0.80 |
| Breastfeeding (y, n, %y) | 122, 1, 99.2% | 72, 1, 98.6% | 0.98 |
| Breastf Freq (≥7, <7, %≥7) | 87, 36, 70.7% | 57, 16, 78.1% | 0.34 |
| Infant Characteristics |  |  |  |
| Age (days) | 32.35 ± 3.11 | 32.86 ± 6.37 | 0.52 |
| Infant Sex (m, f, %m) | 52, 71, 42.3% | 34, 49, 46.6% | 0.17 |
| Weight (kg) | 4.63 ± 0.45 | 4.55 ± 0.56 | 0.31 |
| Length (cm) | 54.36 ± 1.85 | 54.56 ± 2.03 | 0.48 |
| Infant Anthropometrics | | | |
| Total Subcutaneous Fat (mm) | 29.75 ± 5.03 | 28.82 ± 6.34 | 0.29 |
| Central:Total Subc Fat | 0.39 ± 0.04 | 0.38 ± 0.04 | 0.12 |
| Midthigh Skinfold (mm) | 11.57 ± 2.27 | 11.46 ± 3.00 | 0.79 |
| Tricep Skinfold (mm) | 6.50 ± 1.50 | 6.36 ± 1.55 | 0.53 |
| Suprailiac Skinfold (mm) | 4.37 ± 1.23 | 4.21 ± 1.20 | 0.38 |
| Subscapular Skinfold (mm) | 7.31 ± 1.68 | 6.79 ± 1.64 | 0.03 |
| Umbilical Circumference (cm) | 36.05 ± 2.12 | 35.56 ± 2.12 | 0.12 |

**Table S1.** 1-month characteristics of Hispanic mother-infant dyads from the Southern California Mother’s Milk Study are shown for both those included and those excluded from the current study. Data are reported mean and standard deviation (SD) unless otherwise noted. Group means of continuous variables were compared via two-sample *t*-tests and binary variables were compared via Chi-square test. Breastfeeding frequency at 6 months of age is shown dichotomized as ≥7 or <7 times per day. Total subcutaneous fat is the sum of the four infant skinfold thickness measures in millimeters. The Central:Total subcutaneous fat variable is the sum of the suprailiac and subscapular skinfold thicknesses divided by all four skinfold measures.
