# Supplementary material for: Prenatal exposure to ambient air pollutants and early infant growth and adiposity in the Southern California Mother’s Milk Study
Source: Environ Health. 2021 Jun 5;20:67. doi: 10.1186/s12940-021-00753-8 (PMC8180163; doi:10.1186/s12940-021-00753-8)
Supplement: Supplementary file 2 — Additional file 2: Supplemental Table 2. Levels of Ambient Air Pollutant Exposures and Infant Growth Outcomes Across Tertiles of Socioeconomic Status. Means and standard deviations of ambient air pollutant exposure levels and infant growth change variables are presented across tertiles of socioeconomic status (Hollingshead Index). Tertile means of continuous variables were compared via one-way ANOVA and p-values are shown. TSF represents the change in infant total subcutaneous fat, which is the sum of the four infant skinfold thickness measures in millimeters. CTSF represents the change in infant central:total subcutaneous, which is the sum of the suprailiac and subscapular skinfold thicknesses divided by all four skinfold measures. Oxwt is the redox-weight oxidative potential of NO2 and O3. [file 12940_2021_753_MOESM2_ESM.docx]

**Supplemental Table 2. Levels of Ambient Air Pollutant Exposures and Infant Growth Outcomes Across Tertiles of Socioeconomic Status**

|  | **T1 (n=41)**  ***Mean ± SD*** | **T2 (n=41)**  ***Mean ± SD*** | **T3 (n=41)**  ***Mean ± SD*** | **p** |
| --- | --- | --- | --- | --- |
| Prenatal Air Pollutants |  |  |  |  |
| PM_2.5_ (μg/m^3^) | 12.19 ± 1.34 | 11.71 ± 1.04 | 11.95 ± 1.01 | 0.16 |
| PM_10_ (μg/m^3^) | 31.38 ± 3.91 | 30.46 ± 3.44 | 31.26 ± 3.21 | 0.44 |
| NO_2_ (ppb) | 18.19 ± 2.36 | 18.30 ± 2.28 | 17.59 ± 2.37 | 0.35 |
| O_3_ (ppb) | 25.82 ± 2.19 | 25.75 ± 2.42 | 26.53 ± 2.53 | 0.28 |
| O_x_^wt^ | 23.23 ± 0.96 | 23.22 ± 1.04 | 23.49 ± 1.13 | 0.43 |
| Infant Change Variables |  |  |  |  |
| Weight (kg) | 3.36 ± 0.62 | 3.52 ± 0.67 | 3.45 ± 0.62 | 0.51 |
| Length (cm) | 12.69 ± 1.86 | 13.19 ± 1.66 | 12.44 ± 1.73 | 0.15 |
| Umbilical Circ (cm) | 5.44 ± 2.95 | 5.91 ± 2.52 | 5.59 ± 2.55 | 0.72 |
| TSF (mm) | 12.20 ± 8.77 | 13.85 ± 7.06 | 13.32 ± 7.33 | 0.61 |
| CTSF | -0.07 ± 0.04 | -0.07 ± 0.05 | -0.07 ± 0.04 | 0.99 |

**Supplemental Table 2.** Means and standard deviations of ambient air pollutant exposure levels and infant growth change variables are presented across tertiles of socioeconomic status (Hollingshead Index). Tertile means of continuous variables were compared via one-way ANOVA and p-values are shown. TSF represents the change in infant total subcutaneous fat, which is the sum of the four infant skinfold thickness measures in millimeters. CTSF represents the change in infant central:total subcutaneous, which is the sum of the suprailiac and subscapular skinfold thicknesses divided by all four skinfold measures. O_x_^wt^ is the redox-weight oxidative potential of NO_2_ and O_3_.
