# Supplementary material for: Prenatal exposure to ambient air pollutants and early infant growth and adiposity in the Southern California Mother’s Milk Study
Source: Environ Health. 2021 Jun 5;20:67. doi: 10.1186/s12940-021-00753-8 (PMC8180163; doi:10.1186/s12940-021-00753-8)
Supplement: Supplementary file 3 — Additional file 3: Supplemental Table 3. Sensitivity Analysis: Prenatal Exposures and Infant Changes from 1- to 6-Months Postpartum with Adjustment for Area-Level Socioeconomic Status. Multivariable linear regression was performed to examine the relationships between prenatal exposure to ambient air pollutants and changes in infant growth from 1 to 6 months of age with adjustment for a poverty index variable in place of the individual-level socioeconomic status variable in the main model. Beta coefficients and 95% confidence intervals are shown for a one standard deviation increase in exposure (PM2.5 [SD = 1.15 μg/m3], PM10 [SD = 3.54 μg/m3], NO2 [SD = 2.34 ppb], O3 [SD = 2.38 ppb], Oxwt [SD = 1.04 ppb]). Multivariable linear models adjust for infant sex, infant age, pre-pregnancy BMI, breastfeeding frequency, maternal age, and poverty index (a component of the CalEnviroScreen 3.0 score). TSF and CTSF represent changes in total subcutaneous fat and the ratio of central to total subcutaneous fat, respectively. [file 12940_2021_753_MOESM3_ESM.docx]

**Supplemental Table 3. Sensitivity Analysis: Prenatal Exposures and Infant Changes from 1- to 6-Months Postpartum with Adjustment for Area-Level Socioeconomic Status**

| **Exposure** | **Δ Outcome** | **β** | **95% CIs** | **P-value** |
| --- | --- | --- | --- | --- |
| PM_2.5_ | Weight | 0.11 | -0.03, 0.24 | 0.11 |
|  | Length | 0.04 | -0.35, 0.42 | 0.85 |
|  | Umbilical Circ | 0.42 | -0.17, 1.00 | 0.16 |
|  | TSF | 2.28 | 0.60, 3.96 | 0.01 |
|  | CTSF | 0.00 | -0.01, 0.01 | 0.44 |
| PM_10_ | Weight | 0.08 | -0.05, 0.20 | 0.22 |
|  | Length | 0.06 | -0.30, 0.41 | 0.74 |
|  | Umbilical Circ | 0.70 | 0.18, 1.23 | 0.01 |
|  | TSF | 1.61 | 0.04, 3.18 | 0.05 |
|  | CTSF | 0.01 | 0.00, 0.01 | 0.10 |
| NO_2_ | Weight | 0.17 | 0.04, 0.30 | 0.01 |
|  | Length | 0.19 | -0.19, 0.56 | 0.32 |
|  | Umbilical Circ | 0.01 | -0.57, 0.59 | 0.96 |
|  | TSF | 1.92 | 0.27, 3.58 | 0.02 |
|  | CTSF | -0.00 | -0.02, 0.00 | 0.16 |
| O_3_ | Weight | -0.11 | -0.23, 0.01 | 0.08 |
|  | Length | -0.16 | -0.51, 0.18 | 0.35 |
|  | Umbilical Circ | 0.48 | -0.05, 1.01 | 0.07 |
|  | TSF | -0.98 | -2.54, 0.59 | 0.22 |
|  | CTSF | 0.01 | 0.00, 0.02 | 0.02 |
| O_x_^wt^ | Weight | -0.05 | -0.16, 0.07 | 0.42 |
|  | Length | -0.11 | -0.45, 0.22 | 0.50 |
|  | Umbilical Circ | 0.67 | 0.17, 1.16 | 0.01 |
|  | TSF | -0.20 | -1.70, 1.31 | 0.80 |
|  | CTSF | 0.01 | 0.00, 0.02 | 0.02 |

**Supplemental Table 3.** Multivariable linear regression was performed to examine the relationships between prenatal exposure to ambient air pollutants and changes in infant growth from 1 to 6 months of age with adjustment for a poverty index variable in place of the individual-level socioeconomic status variable in the main model. Beta coefficients and 95% confidence intervals are shown for a one standard deviation increase in exposure (PM_2.5_ [SD=1.15 ug/m3], PM_10_ [SD=3.54 ug/m3], NO_2_ [SD=2.34 ppb], O_3_ [SD=2.38 ppb], O_x_^wt^ [SD=1.04 ppb]). Multivariable linear models adjust for infant sex, infant age, pre-pregnancy BMI, breastfeeding frequency, maternal age, and poverty index (a component of the CalEnviroScreen 3.0 score). TSF and CTSF represent changes in total subcutaneous fat and the ratio of central to total subcutaneous fat, respectively.
