# Supplementary material for: Prenatal exposure to ambient air pollutants and early infant growth and adiposity in the Southern California Mother’s Milk Study
Source: Environ Health. 2021 Jun 5;20:67. doi: 10.1186/s12940-021-00753-8 (PMC8180163; doi:10.1186/s12940-021-00753-8)
Supplement: Supplementary file 4 — Additional file 4: Supplemental Table 4. Sensitivity Analysis: Prenatal Exposures and Infant Z-Score Changes from 1- to 6-Months Postpartum. Table displays the results for multivariable linear regression models examining prenatal ambient air pollutant exposure and changes in infant z-score measures (weight-for-age and weight-for-length). Beta coefficients are shown for a one standard deviation increase in exposure (PM2.5 [SD = 1.15 μg/m3], PM10 [SD = 3.54 μg/m3], NO2 [SD = 2.34 ppb], O3 [SD = 2.38 ppb], Oxwt [SD = 1.04]). Models adjusted for pre-pregnancy BMI, breastfeeding frequency, maternal age, and socioeconomic status. [file 12940_2021_753_MOESM4_ESM.docx]

**Supplemental Table 4. Sensitivity Analysis: Prenatal Exposures and Infant Z-Score Changes from 1- to 6-Months Postpartum**

| **Exposure** | **Δ Outcome (z-scores)** | **β** | **p-value** |
| --- | --- | --- | --- |
| PM_2.5_ | Weight-for-length | 0.23 | 0.01 |
|  | BMI-for-age | 0.07 | 0.37 |
| PM_10_ | Weight-for-length | 0.04 | 0.14 |
|  | BMI-for-age | -0.00 | 0.93 |
| NO_2_ | Weight-for-length | 0.12 | 0.01 |
|  | BMI-for-age | 0.06 | 0.13 |
| O_3_ | Weight-for-length | -0.08 | 0.07 |
|  | BMI-for-age | -0.06 | 0.16 |
| O_x_^wt^ | Weight-for-length | -0.08 | 0.44 |
|  | BMI-for-age | -0.09 | 0.31 |

**Supplemental Table 4.** Table displays the results for multivariable linear regression models examining prenatal ambient air pollutant exposure and changes in infant z-score measures (weight-for-age and weight-for-length). Beta coefficients are shown for a one standard deviation increase in exposure (PM_2.5_ [SD=1.15 ug/m3], PM_10_ [SD=3.54 ug/m3], NO_2_ [SD=2.34 ppb], O_3_ [SD=2.38 ppb], O_x_^wt^ [SD=1.04 ppb]). Models adjusted for pre-pregnancy BMI, breastfeeding frequency, maternal age, and socioeconomic status.
