# Supplementary figures and images for: Prenatal exposure to ambient air pollutants and early infant growth and adiposity in the Southern California Mother’s Milk Study
Source: Environ Health. 2021 Jun 5;20:67. doi: 10.1186/s12940-021-00753-8 (PMC8180163; doi:10.1186/s12940-021-00753-8)

**Supplemental Figure 1. Flowchart Detailing Participants Retained for the Present Analysis**

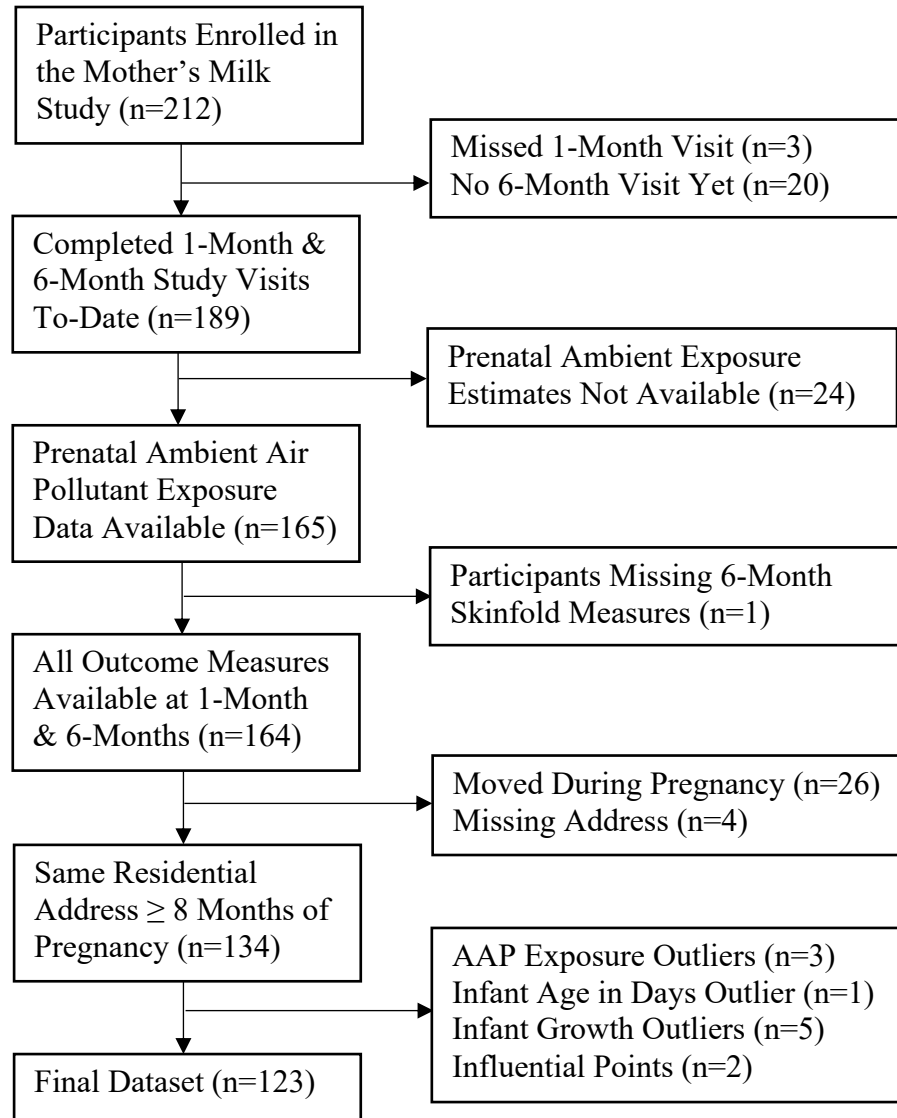

Supplement: Supplementary file 5 — Additional file 5: Supplemental Figure 1. Flowchart Detailing Participants Retained for the Present Analysis. At the time of our analyses, 212 participants had been enrolled in the Mother’s Milk Study. There were 24 participants who had completed both clinical study visits but did not yet have ambient pollutant exposure estimates available. Among the 5 change in infant growth variable outliers, 2 were outliers from infant length change, 1 was an outlier from infant weight change, 1 was an outlier from umbilical circumference change, and 1 was from change in CTSF. [file 12940_2021_753_MOESM5_ESM.pdf]
