# Supplementary material for: Prenatal exposure to ambient air pollutants and early infant growth and adiposity in the Southern California Mother’s Milk Study
Source: Environ Health. 2021 Jun 5;20:67. doi: 10.1186/s12940-021-00753-8 (PMC8180163; doi:10.1186/s12940-021-00753-8)

Supplemental Figure 2. Sensitivity Analysis: Multivariable Linear Models Additionally Adjusted for Birth Weight and Baseline of Change Variables

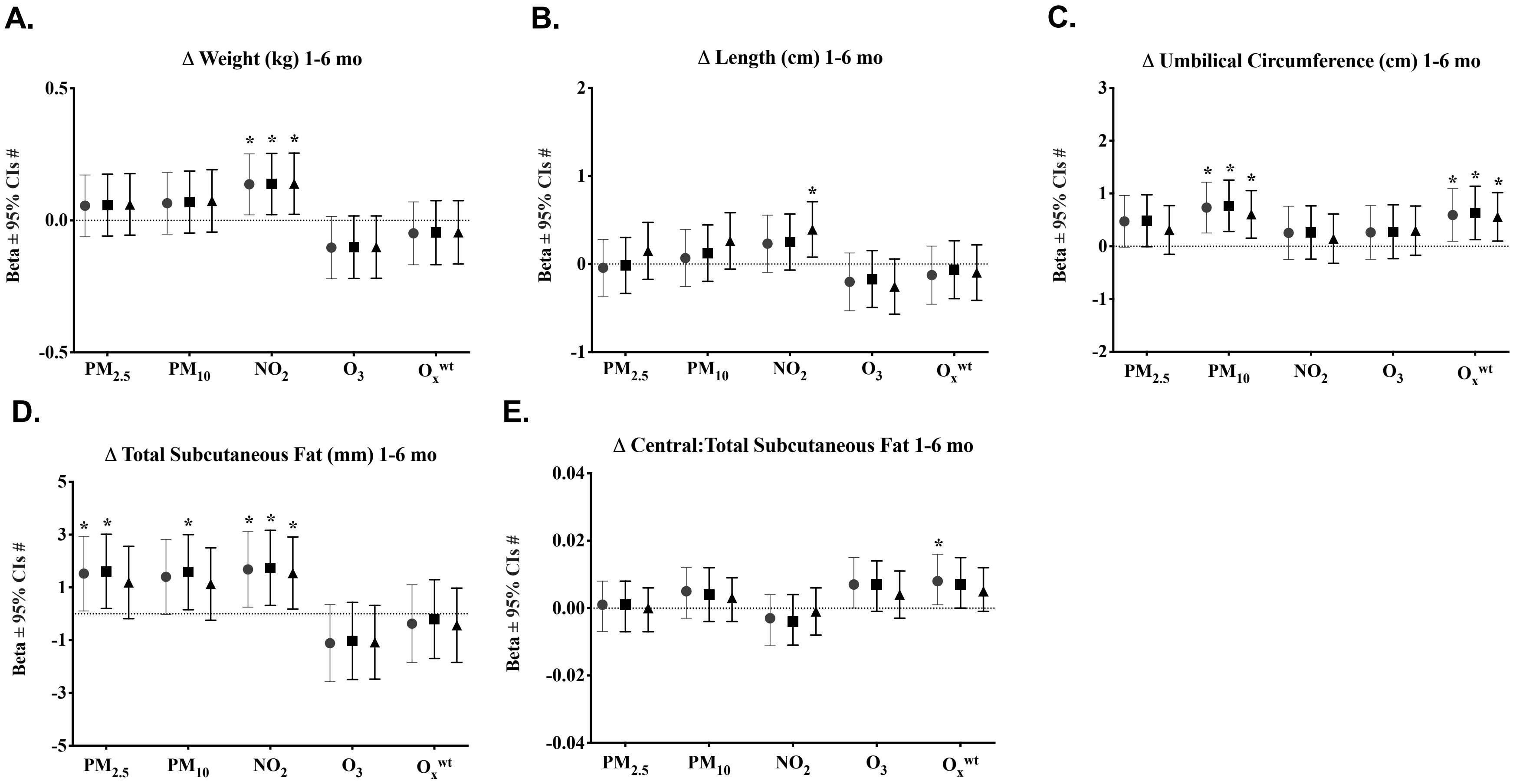

Supplement: Supplementary file 6 — Additional file 6: Supplemental Figure 2. Sensitivity Analysis: Multivariable Linear Models Additionally Adjusted for Birth Weight and Baseline of Change Variables. Fully adjusted models controlled for infant sex, infant age, pre-pregnancy BMI, breastfeeding frequency, maternal age, and socioeconomic status (effect estimates denoted by circles). Sensitivity analyses examining multivariable linear regression models additionally adjusting for birth weight (effect estimates denoted by squares) or the baseline value of the change variable (effect estimates denoted by black triangles), respectively, were performed to further evaluate the relationships between prenatal exposure to ambient air pollutants and changes in infant growth from 1 to 6 months of age. Beta coefficients and 95% confidence intervals are shown for a one standard deviation increase in exposure (PM2.5 [SD = 1.15 μg/m3], PM10 [SD = 3.54 μg/m3], NO2 [SD = 2.34 ppb], O3 [SD = 2.38 ppb], Oxwt [SD = 1.04]). [file 12940_2021_753_MOESM6_ESM.pdf]
