# Supplementary material for: Prenatal exposure to ambient air pollutants and early infant growth and adiposity in the Southern California Mother’s Milk Study
Source: Environ Health. 2021 Jun 5;20:67. doi: 10.1186/s12940-021-00753-8 (PMC8180163; doi:10.1186/s12940-021-00753-8)

Supplemental Figure 3. Scatterplots Displaying Change in Umbilical Circumference in response to Prenatal NO_2 and O_3

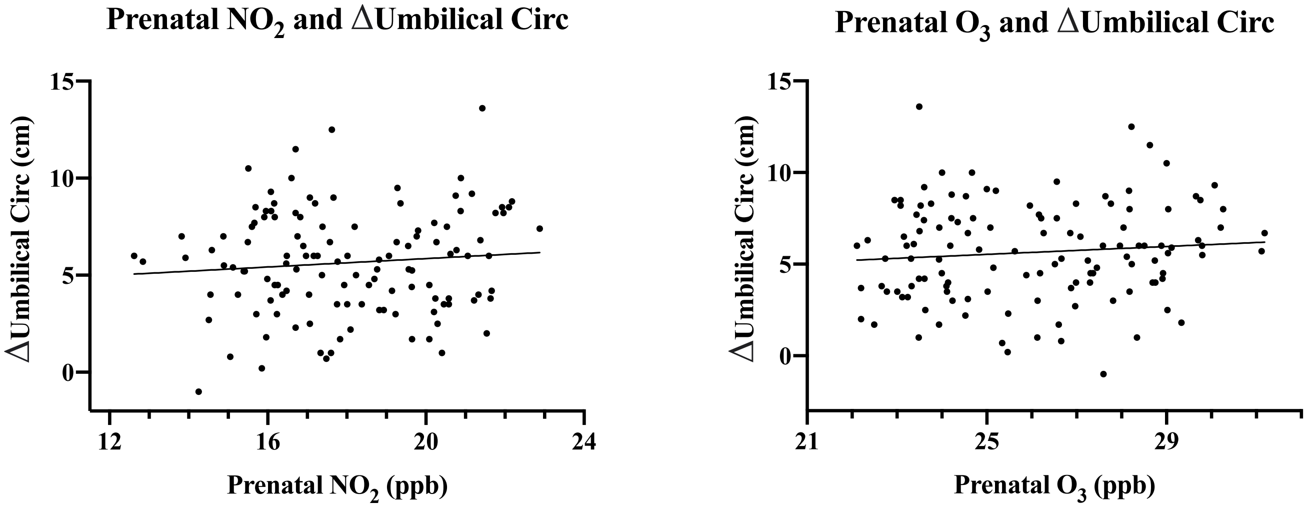

Supplement: Supplementary file 7 — Additional file 7: Supplemental Figure 3. Prenatal NO2 and O3 were Associated with a Greater Change in Umbilical Circumference. Scatterplots display infant change in umbilical circumference in relation to prenatal NO2 and O3, respectively. Plots were made to visualize the distribution of these data due to a strong inverse correlation between NO2 and O3, yet similar direction of effect. [file 12940_2021_753_MOESM7_ESM.pdf]
